# Whole-genome sequencing revealed a novel structural variant in *COL4A4* causing autosomal dominant Alport syndrome: A case report

**DOI:** 10.1016/j.heliyon.2024.e40802

**Published:** 2024-11-28

**Authors:** Clément Delage, Marine Andreani, Nihad Boukrout, Naoual Sabaouni, Michaël Perrais, Bruno Lefebvre, Christelle Cauffiez, Nicolas Pottier, Romain Larrue

**Affiliations:** a Service de Toxicologie et Génopathies, CHU Lille, F-59000, Lille, France; b Service de Néphrologie, CHU de Nice, F-06000, Nice, France; c Univ. Lille, CNRS, Inserm, CHU Lille, Institut Pasteur de Lille, UMR9020-U1277-CANTHER-Cancer Heterogeneity Plasticity and Resistance to Therapies, F-59000, Lille, France

**Keywords:** Case report, Sequencing, Alport diseases, Genetic variations

## Abstract

Next-generation sequencing has substantially transformed the genomic diagnosis of individuals affected by inherited renal disorders. Indeed, accurate and rapid diagnostic for patients with suspected genetic kidney diseases is not only important for prognosis and patient management but also for family counseling. Alport syndrome, a genetic disease primarily affecting the basement membrane, is characterized by hematuria, progressive kidney failure, hearing impairment, as well as ocular abnormalities and stems from mutations in genes encoding type IV collagen. In this study, we show the benefit of whole-genome sequencing for the molecular diagnosis of a dominant form of Alport syndrome by identifying a novel heterozygous pathogenic structural variant in a family with three affected members. This case underscores the potential of whole-genome sequencing as a frontline diagnostic approach for inherited kidney diseases and further indicates that structural variations represent an important cause of monogenic disorders.

## 1 Introduction

Alport syndrome (AS) is a rare form of inherited collagenopathy with an estimated prevalence of approximately 1 in 50,000 live births and is characterized by a structural disorder of the basement membrane (BM) [1,2]. This genetic disease is defined by the appearance of nephropathy secondary to progressive glomerulonephritis, high tone sensorineural deafness and ocular abnormalities including anterior lenticonus or retinal flecks [3,4]. Type IV collagen is the main collagen component of the BM and provides structural and functional support to neighboring cells [5]. Type IV collagen derives from six genetically distinct polypeptide chains, named α1(IV) through α6(IV), that share between 50 % and 70 % homology [5]. Previous biochemical studies have notably shown that three α-chains assemble into heterotrimeric molecules that further associate to form supramolecular networks, defining the type IV collagen [5,6]. α1(IV) and α2(IV) chains are ubiquitously expressed in most BMs whereas the remaining α-chains display more restricted tissue distribution [5,6]. For example, the α3(IV)/α4(IV)/α5(IV) network is found mainly in the mature glomerular BM (GBM) [5].

AS arises from mutations in type IV collagen genes. Mutations in the X-linked *COL4A5* gene (MIM#301050) are found in approximately 80 % of cases while the remaining 20 % present genetic alterations in the autosomal *COL4A3* or *COL4A4* genes and are transmitted according to a dominant (MIM#104200) or recessive (MIM#203780) mode of inheritance [7]. Given that all three type IV collagen α-chains are necessary to form the heterotrimer, deleterious genetic variations in any of these type IV collagen genes result in the complete or partial loss of the α3(IV)/α4(IV)/α5(IV) network [5]. As consequences, mutations found in AS patients lead to severe GBM ultrastructural abnormalities, progressive scarring of glomeruli and impaired kidney function [5,8]. Genetic testing is an essential step in the clinical management of AS, not only because the clinical features, family history, and even kidney biopsy are often not sufficient for accurate diagnosis but also because it informs on the mode of inheritance and, sometimes, the likelihood of early-onset kidney failure and extrarenal manifestations [2].

In this study, we demonstrated, for the first time, the clinical relevance of applying whole-genome sequencing as a first-line diagnostic tool for AS and further highlighted that structural variations represent an important cause of AS.

## 2 Case report

**Fig. 1:**
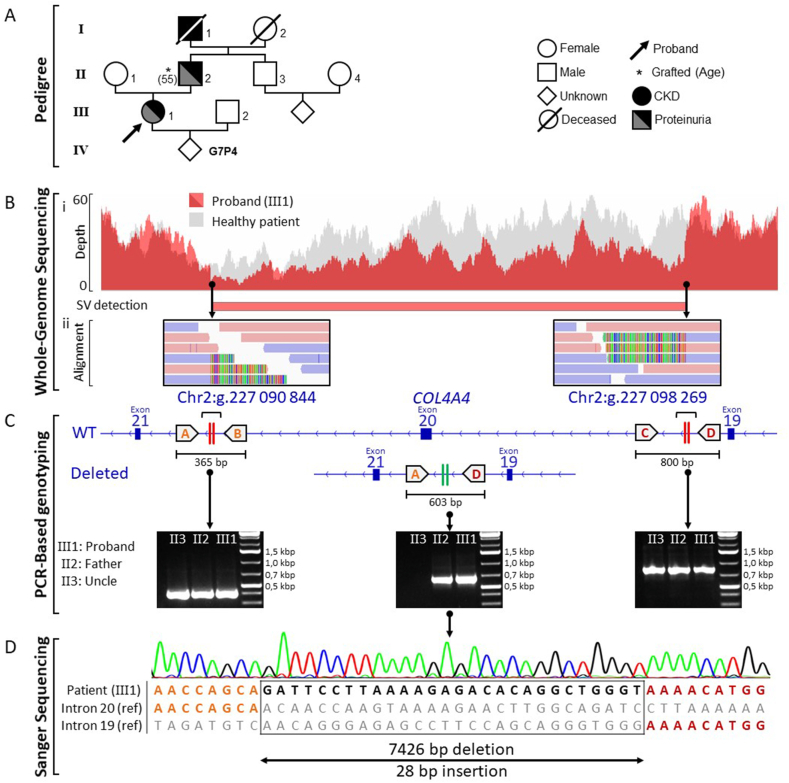
**Identification and characterization of a novel structural variant affecting the *COL4A4* gene. (A)** Pedigree of family. **(B)** Identification of the structural variant by whole-genome sequencing. (Bi) Read depth plot indicating in the proband the heterozygous ∼7.4 kb deletion comprising exon 20 and its surrounding region within the *COL4A4* gene in comparison with a healthy individual. (Bii) Integrated Genome Viewer screenshots showing breakpoint sequence alignments. Soft-clipped reads (appearing as multicolor rainbow bands) derive from the deleted allele and delineate the exact breakpoint positions whereas fully mapped reads derive from the wild-type allele (plus strand reads are shown in pink and minus strand reads in blue). **(C)** PCR-based genotyping strategy used to discriminate between the deleted allele and the wild type allele. Four primers (**A–D**) were designed and three allele-specific PCR including two PCR spanning the deletion breakpoints (indicated by two vertical red stripes) and one PCR surrounding the fusion point between intron 19 and 20 (indicated by two vertical green stripes) were performed using DNA from the proband and his affected father as well as DNA from his healthy uncle. **(D)** Sanger sequencing of the PCR products obtained with the primer A and D was performed to characterize the deleted allele of the proband.

We describe the case of a 42-year-old female (Fig. 1A, patient III1) presenting with hematuria, proteinuria, stage 3b chronic kidney disease (CKD) (creatinine = 164 μmol/L, eGFR = 30 mL/min/1,73 m^2^), blood hypertension, dyslipidemia and one renal cyst (2 cm). The patient was referred to a nephrologist at age of 37 after the discovery of a proteinuria quantified at 2.61 g/24h and a renal biopsy was performed. Analysis of the kidney section by light microscopy revealed non-specific findings including interstitial fibrosis, tubular atrophy as well as glomerular lesions such as irregular thickening of Bowman's capsule. However, typical structural abnormalities associated with AS (splitting and lamellation of the GBM lamina densa) was found using electron microscopy [9]. Since then, the patient's renal function showed a progressive decline despite nephroprotective measures such as the use of renin–angiotensin system inhibitor. A detailed review of family history (Fig. 1A) revealed that the father (II2) and the paternal grandfather (I1) also experienced CKD of unknown origin. The father (II2) received dialysis at age 50 until he was transplanted at the age of 55 whereas the grandfather (I4) died at the age of 31 from renal failure.

A peripheral blood sample was obtained from the proband (III1) and two relatives (II2 and II3), followed by genomic DNA extraction. Initial genetic testing for AS was performed using a dedicated targeted Next-generation sequencing (NGS) panel specifically designed to capture the coding regions and the exon–intron boundaries of the *COL4A* genes implicated in AS (HaloPlex-based target enrichment). Analysis of the sequencing data (short-read sequencing) showed a total of 85 exonic and near splice site variants, but none of them was judged clinically relevant based on frequency and *in silico* analysis. Given the proband clinical features reminiscent of AS, WGS was performed. Indeed, this technology enables the identification of nearly all forms of genetic variations, including disease-causing deep intronic mutations and structural variants, that are largely missed by capture sequencing-based technologies targeting the coding regions [10,11]. This analysis led to the identification of a novel non-recurrent heterozygous 7,4 kb deletion (chr2:227090844:L7426:DEL) surrounding *COL4A4* exon 20 and categorized as “likely pathogenic” according to the recommendation of the American College of Medical Genetics and Genomics (ACMG) and the Clinical Genome Resource (ClinGen) [12]. The deletion was detected by comparing read depth to a healthy individual and by examining the sequence of partially misaligned reads (Fig. 1B). No additional pathogenic variant was detected. PCRs (Table 1) and Sanger sequencing were then carried out to confirm the NGS result (Fig. 1C–D). This variant was also identified by PCR in the proband's father (II2), indicating paternal transmission (Fig. 1C). Finally, the uncle (II3) of the proband (III1) showed no abnormalities in the *COL4A4* gene, which was consistent with the clinical data (Fig. 1C). Overall, the proband was finally diagnosed with autosomal dominant AS, and the 7.4 kb deletion of chromosome 2 was the cause of the disease.

**Table 1:** List of the primers.

Primer	Localisation	Sequence (5′-3′)	Tm (°C)
A	Intron 20	AGTTTGGCATCCTCCCCAAAT	63.9
B		GCAGGCTGTTCATGTTTCAGG	63.0
C	Intron 19	AGGACTCTTTCCCAGGCTTTTT	61.7
D		TGCACCCCAGAACATTCAATC	63.1

## 3 Discussion

Rare inherited diseases, though individually uncommon, collectively affect nearly 300 million individuals worldwide and thus represent a significant public health challenge [13]. Advances in NGS technologies have substantially transformed molecular diagnosis practices, driving a shift from targeted genetic testing toward sequencing an entire exome or genome [14]. Among these, WGS stands out as the most comprehensive sequencing approach, allowing the detection of all types of genetic alterations in a single-laboratory workflow [14]. In this case study, we highlight the advantages of WSG as a first-line diagnostic tool, particularly for inherited kidney disorders such as Alport syndrome, and further show that this approach is also of great interest in cases with strong clinical suspicion of a particular disease where complex structural variants are likely or prior inconclusive targeted sequencing.

AS is a mendelian disease and ranks as the second most common monogenic cause of chronic kidney diseases after autosomal dominant polycystic kidney disease [15]. Suspicion of AS mostly relies on clinical and paraclinical findings as well as family history [15]. Although histological and ultrastructural analyses of the kidney biopsies represent a valuable tool for distinguishing AS from other inherited kidney disorders, genetic testing is currently the only definitive method to unambiguously identify AS, establishing thus an unequivocal diagnosis for patients and families [15]. Nevertheless, molecular analyses of genetically heterogeneous diseases such as AS is still challenging even when applying standard NGS methods [10]. Indeed, targeted sequencing of the coding regions and adjacent splice sites exhibits several technical limitations, especially for the detection of complex structural variants which are common in AS [10,15]. In line with this, the initial targeted sequencing approach did not retrieve any relevant variant despite strong clinical evidence of AS in the studied proband, whereas a comprehensive analysis using WGS identified a causative large deletion in *COL4A4*. Genomic deletions usually occur during double strand break DNA repair through non-replicative repair mechanisms such as non-homologous end-joining or replicative-based repair mechanisms including fork stalling and template switching [[16], [17], [18]]. Noteworthy, our analysis revealed the insertion of a 28 bp sequence at the breakpoint, a phenomenon typically associated with imprecise repair mechanisms, in particular non-homologous end-joining [[16], [17], [18]].

## 4 Conclusion

In conclusion, this case report demonstrates that WGS provides a comprehensive and efficient diagnostic approach for AS, increasing diagnostic yields, reducing diagnostic delays and improving patient management.

## CRediT authorship contribution statement

**Clément Delage:** Writing – review & editing. **Marine Andreani:** Resources. **Nihad Boukrout:** Formal analysis. **Naoual Sabaouni:** Investigation. **Michaël Perrais:** Writing – review & editing. **Bruno Lefebvre:** Writing – review & editing. **Christelle Cauffiez:** Writing – review & editing, Supervision. **Nicolas Pottier:** Writing – original draft, Conceptualization. **Romain Larrue:** Formal analysis.

## Ethics statement

This study complies with French laws and has been approved by the Institutional Review Board of Centre Hospitalier Universitaire de Lille (France). All participants provided written informed consent for genetic analysis and publication of this study in line with institutional guidelines and the Declaration of Helsinki and Istanbul. The DNA collection is officially registered with the “Ministère de l’Enseignement Supérieur et de la Recherche (Paris, France)” under the number: DC-2008–642.

## Data availability statement

Data will be made available on request.

## Declaration of competing interest

The authors declare that they have no known competing financial interests or personal relationships that could have appeared to influence the work reported in this paper.
